# Radiographic Assessment of Permanent First Molars Among Adults in Riyadh: A Retrospective Study

**DOI:** 10.7759/cureus.33336

**Published:** 2023-01-04

**Authors:** Reema S Alshawaf, Asma A Alhussain, Hafsah H Al Ali, Azizah A Al-mutairi, Noura F AlRebdi

**Affiliations:** 1 Oral Medicine and Diagnostic Sciences, College of Dentistry, King Saud University, Riyadh, SAU; 2 Dentistry, College of Dentistry, King Saud University, Riyadh, SAU

**Keywords:** fpm, assessment, status, radiographic assessment, panoramic, loss, missing, first permanent molars

## Abstract

Introduction

The first permanent molars (FPMs) are the first permanent teeth to emerge in the oral cavity, which have an important role in dentition, dental development, and occlusion. FPMs are the most susceptible teeth to caries due to early eruption. There are many adverse consequences related to losing FPMs. Many of the previous studies focused on younger age groups. To the best of our knowledge, there needs to be more newly updated data reported in the literature regarding the status of FPMs in adults in Saudi Arabia. The study aimed to assess the status of permanent first molars among adults in a sample of patients in Riyadh.

Methods

A cross-sectional retrospective study was conducted from the records of patients in the Dental University Hospital, College of Dentistry, King Saud University (KSU), after obtaining ethical approval. Panoramic images of 810 patients ages 18 and above who were seen at KSU, College of Dentistry, were examined to assess the status of FPMs. A panoramic examination was undertaken for various dental reasons in the time between the years 2017 and 2022. Patients were divided into three age groups: 18-30, 31-50, and >50 years. Data were collected in a particular form and analyzed. The prevalence of missing teeth, as well as restored and carious teeth, were recorded. In addition, further information regarding the type of restoration was recorded. Correlations of the findings regarding age, gender, side, and jaw were determined for all cases and were performed using the chi-square test. P-values less than 0.05 were considered statistically significant.

Results

A total of 810 patients' panoramic radiographs were studied. Panoramic male study subjects were 382 (47.2%), and females were 427 (52.8%). Three-hundred thirty-nine (339; 41.9%) of them were of less than 30 years of age, 327 (40.4%) were in the age group of 31-50 years, and the remaining 144 (17.8%) were above 50 years of age. The prevalence of missing first molar teeth was 145 (17.9%) for teeth 16, 147 (18.1%) for teeth 26, 207 (25.5%) for teeth 36, and 188 (23.2%) for teeth 46. No statistically significant association was found between the age groups and gender and the first molar missing teeth (p=0.848 & p=0.159). Nineteen point thirteen percent (19.13%) of patients had only one missing FPM, 12.3% of patients had two, and 6.29% of them had three missing FPM teeth. Five point fifty-five percent (5.55%) of patients had four missing FPMs. A total of 56.8% (242) radiographs in the overall sample did not show any missing teeth. The mandibular FPMs (35.2%) were found missing more frequently than their maxillary counterparts (14.8%), and the left mandibular quadrant showed a slightly higher rate of missing FPM (25.55%). Statistically, a significant association was seen between the number of missing FPM among different age groups, p-value <0.0001. The most restored, carious, and replaced tooth was the mandibular left molar, and tooth-color restoration was the most commonly used restoration.

Conclusion

A high prevalence rate of missing first permanent molars (FPMs) was found in this study population. This calls for more awareness and preventive measures to preserve such significant teeth, therefore reducing the need for complex procedures. Further prospective studies on a larger population are needed to comprehensively evaluate the status of FPMs.

## Introduction

The first permanent molars (FPMs) are the first teeth to emerge in the oral cavity, which have an important role in dentition, dental development, and occlusion. FPMs are the main occluding areas in the mandible [1]. Therefore, they have an essential role in orthodontics, as the anchorage in tooth movement. In addition, a missing first molar will complicate the orthodontic treatment as closing long edentulous spaces increases treatment time, and good outcomes might be difficult to achieve [2]. Other problems may also include reduced masticatory efficiency, esthetic problems, and malalignment of adjacent teeth.

Unfortunately, first permanent molars are the highest susceptible teeth to caries due to their early eruption [3]. Caries was found to be the main reason for permanent tooth extraction [4,5]. Previous studies in Saudi Arabia have found an 83% prevalence of caries among children, and the prevalence of missing FPMs was higher with mandibular FPMs than the maxillary FPMs [6,7]. However, older adults face many health problems, which put them at a higher risk of caries [8]. Therefore, studies are required to determine the status of the first permanent molars and associated factors to help plan preventive and educational programs and apply early dental interventions to avoid complex treatments.

To the best of our knowledge, little newly updated data are reported in the literature regarding the status of permanent first molars in Saudi Arabia, especially among adults.

Therefore, this study aimed to assess the coronal status of the first permanent molars in university clinic patients, to find the rates of missing, restored, or carious and endodontically treated first molars, and to compare the status of first permanent molars with patient demographics (age, gender).

## Materials and methods

This cross-sectional retrospective study was conducted at the College of Dentistry, King Saud University, Saudi Arabia. The study protocol was approved by the Committee of Ethics in Research at the College of Dentistry Research Center and was conducted in compliance with the "Ethical principles for medical research involving human subjects" of the Declaration of Helsinki. The consent of patients to use data was obtained as part of their agreeing to be treated in the College of Dentistry.

A sample set of panoramic radiographs (from 2017 to 2022) was obtained from patients' digital records. All the panoramic radiographs included in this study were obtained from patients aged 18 years and above. Radiographs with severe overlapping or any other cause that resulted in an unreliable diagnosis were excluded from the study sample.

A total of 810 digital panoramic images were included in this study. Panoramic images were previously taken using Planmeca ProMax (Planmeca, Helsinki, Finland). Demographic information, including gender and age, was obtained from the patient’s records. Panoramic images were examined for the evaluation of FPMs. Information regarding whether the first permanent molar was present or missing was recorded. Missing FPM teeth in both the maxillary and mandibular arches on the right or left side of the arch were determined from panoramic radiographs as well as the number of missing teeth. Information regarding whether the tooth was replaced by an implant or pontic was also recorded.

In the case of the present FPM, information regarding whether the tooth was sound, carious, or restored was gathered. The type of restoration was recorded, as well as if the tooth had root canal treatment (RCT) or not. In the case of RCT information regarding whether the tooth was crowned or restored was obtained. Our study followed the Fédération Dentaire Internationale (FDI) numbering system referring to the first molars (#16,26,36 and 46). Whereas #16 is the right maxillary FPM, #26 is the maxillary left FPM, #36 is the mandibular left FPM, and #46 is the mandibular right FPM. Patients were also categorized into three age groups: from 18 to 30, 31 to 50, and more than 50 years old.

Data were analyzed using SPSS (Statistical Package for Social Sciences) version 26.0 software (IBM Inc., Armonk, NY). Descriptive statistics (frequencies and percentages) were used to describe the categorical variables. Pearson's chi-square test was used to compare the distribution of categorical outcome variables (missing teeth, present teeth, sound caries, restored, type of restoration, and remaining root) across the three age groups and gender of study subjects. A p-value of ≤ 0.05 was used to report the statistical significance of the results.

## Results

A total of 810 patients' panoramic radiographs were studied, 339 (41.9%) of them were less than 30 years of age, 327 (40.4%) were in the age group of 31-50 years, and the remaining 144 (17.8%) were above 50 years of age. The male study subjects were 382 (47.2%) and the females were 427 (52.8%). The prevalence of missing first molar teeth was 145 (17.9%) for teeth 16, 147 (18.1%) for teeth 26, 207 (25.5%) for teeth 36, and 188 (23.2%) for teeth 46. The distribution of missing teeth in relation to age groups and gender is shown in Table 1, where no statistically significant association was found between the characteristics of patients (age groups and gender) and the first molar missing teeth (p=0.848 & p=0.159), respectively.

**Table 1:** Prevalence of missing teeth among study subjects in relation to age groups and gender

Study variables	Missing 16 (n=145)	Missing 26 (n=147)	Missing 36 (n=207)	Missing 46 (n=188)	Χ^2^-value	p-value
Age groups <=30	20 (13.8)	28 (19.0)	38 (18.4)	31 (16.5)	2.681	0.848
31-50	68 (46.9)	65 (44.2)	100 (48.3)	88( 46.8)		
>50	57 (39.3)	54 (36.7)	69 (33.3)	69 (36.7)		
Gender Male	64 (44.1)	76 (51.7)	90 (43.5)	74 (39.4)	5.180	0.159
Gender Female	81 (55.9)	71 (48.3)	117 (56.5)	114 (60.6)		

The prevalence of the number of missing teeth and its distribution in relation to age groups and gender is given in Table 2. There is a highly statistically significant association between the age groups of subjects and the prevalence of the number of missing teeth. Thirty-one percent (31%) of subjects who were less than or equal to 30 years had one tooth missing; this is more significant than the proportion of two, three, and four teeth missing in the same age group. In addition, the proportion of four teeth missing is significantly higher (51.1%) in subjects of age group > 50 years when compared with the proportion of one, two, and three teeth missing in the same age group. No statistically significant association was observed between gender and the prevalence of a number of missing teeth (Table 2).

**Table 2:** Prevalence of the number of missing teeth among study subjects in relation to age groups, and gender

Study variables	One missing FPM (n=155)	Two missing FPMs (n=99)	Three missing FPMs (n=51)	Four missing FPMs (n=45)	No missing FPMs (n=460)	Total	Χ^2^-value	p-value
Age groups <=30	48 (31.0)	17 (17.2)	6 (11.8)	4 (8.9)	264 (57.4)	339 (41.9)	162.83	<0.0001
31-50	81 (52.3)	45 (45.5)	26 (51.0)	18 (40.0)	157 (34.1)	327 (40.4)		
>50	26 (16.8)	37 (37.4)	19 (37.3)	23 (51.1)	39 (8.5)	144 (17.8)		
Gender Male	66 (42.6)	47 (47.5)	24 (47.1)	18 (40.0)	227 (49.3)	382 (47.2)	3.12	0.538
Gender Female	89 (57.4)	52 (52.5)	27 (52.9)	27 (60.0)	233 (50.7)	428 (52.8)		

The prevalence of missing teeth in relation to the dental arch (maxilla and mandible) and in relation to the side (right and left) is given in Table 3 and Table 4. The prevalence of one missing and two missing teeth are significantly higher in the mandible (19.1% & 14.8%) when compared with the proportion of one and two missing teeth (16.4% and 9.8%) in the maxilla dental arch (p=0.0001). Furthermore, the prevalence of missing teeth is not statistically significantly different in relation to the side of the dental arch.

**Table 3:** Prevalence of teeth missing in relation to the dental arch (n=810)

No. of teeth	Maxilla	Mandible	Χ^2^-value	p-value
One missing	133 (16.4)	155 (19.1)	13.63	0.001
Two missing	79 (9.8)	120 (14.8)		
No missing	598 (73.8)	535 (66.0)		

**Table 4:** Prevalence of teeth missing in relation to the side (n=810)

No. of teeth	Right	Left	Χ^2^-value	p-value
One missing	186 (23.0)	202 (24.9)	1.05	0.590
Two missing	73 (9.0)	76 (9.4)		
No missing	551 (68.0)	532 (65.7)		

The status of all the first molars (16, 26, 36 46) was assessed, where the prevalence of sound teeth for 16, 26, 36, and 46 were found to be 25.6%, 22.7%, 18.1%, and 18.1%; caries was 14.6%, 15.2%, 11.9% & 11.7%; and restored teeth were 50.7%, 51.4%, 53.5%, and 54,6%, respectively. Table 5 shows the distribution of prevalence of sound, caries, and restoration status of the four first molar teeth across the three age groups and gender, where no statistically significant association between the age groups, gender of patients, and the three characteristics of tooth status (sound, carious, and restored) of four molar teeth (Table 5).

**Table 5:** Prevalence of teeth status among the study subjects in relation to age groups and gender

Study variables	Sound						Caries				Restored			
	16 (n=207)	26 (n=184)	36 (n=147)		46 (n=147)		16 (n=118)	26 (n=123)	36 (n=96)	46 (n=95)	16 (n=411)	26 (n=416)	36 (n=443)	46 (n=442)
Age groups <=30	122 (58.9)	105 (57.1)	88 (59.9)		84 (57.1)		65 (55.1)	64 (52)	48 (34.8)	43 (45.3)	160 (38.9)	161 (38.7)	184 (42.5)	196 (46.1)
31-50	62 (30.0)	56 (30.4)	42 (28.6)		47 (32.0)		40 (33.9)	49 (39.8)	38 (39.6)	43 (45.3)	178 (30.4)	179 (43.0)	179 (41.3)	175 (39.6)
>50	23 (11.1)	23 (12.5)	17 (11.6)		16 (10.9)		13 (11.0)	10 (8.1)	10 (10.4)	9 (9.5)	73 (12.5)	76 (18.3)	70 (10.9)	1 (16.1)
Χ^2^-value; p-value	0.682; 0.995						3.367; 0.762				4.229; 0.646			
Gender Male	114 (55.1)	108 (58.7)		83 (56.5)		90 (61.2)	63 (53.4)	60 (48.8)	50 (52.1)	56 (58.9)	175 (58.7)	165 (39.7)	190 (43.9)	189 (42.8)
Gender Female	93 (44.9)	76 (41.3)		64 (43.5)		57 ( 38.8)	55 (46.6)	63 (51.2)	46 (47.9)	39 (41.1)	236 (57.4)	251 (60.3)	243 (56.1)	253 (57.2)
Χ^2^-value; p-value	1.500; 0.682						2.268; 0.519				1.678;0.642			

The type of restoration out of the number of restored teeth for teeth numbers 16, 26, 36, and 46 is given in Table 6. 

**Table 6:** Prevalence of the type of restoration out of the number of restored teeth

Type of restoration	16 (n=411)	26 (n=416)	36 (n=433)	46 (n=442)
Amalgam	82 (20.0)	77 (18.5)	108 (24.9)	88 (19.9)
Tooth-colored	213 (51.8)	232 (55.8)	221 (51.0)	226 (51.1)
Crown	73 (17.8)	69 (16.6)	63 (14.5)	87 (19.7)
Implant	28 (6.8)	19 (4.6)	23 (5.3)	31 (7.0)
Pontic	11 (2.7)	17 (4.1)	16 (3.7)	7 (1.6)
Amalgam & tooth-colored	4 (1.0)	2 (0.5)	2 (0.5)	2 (0.5)
Χ^2^-value; p-value	440.72; <0.0001	523.92; <0.0001	471.34; <0.0001	476.05; <0.0001

The prevalence of crowned or restored teeth out of the number of RCT teeth and the prevalence of replaced teeth out of missing teeth for each of the four molar teeth (16, 26, 36, and 46) are given in Table 7 and Table 8.

**Table 7:** Prevalence of restored and crowned teeth out of the number of RCT teeth RCT: root canal treatment

	16 (n=124)	26 (n=123)	36 (n=139)	46 (n=144)
Restored teeth	111 (89.5)	107 (87.0)	123 (88.5)	125 (86.8)
Crowned teeth	58 (46.8)	59 (48)	56 (40.3)	68 (47.2)

**Table 8:** Prevalence of replaced teeth out of the number of missing teeth

Replaced teeth	16 (n=145)	26 (n=147)	36 (n=207)	46 (n=188)	Χ^2^-value	p-value
Yes	39 (26.9)	37 (25.2)	39 (18.8)	39 (20.7)	4.14	0.247
No	106 (73.1)	110 (74.8)	168 (81.2)	149 (79.3)		

## Discussion

The present retrospective study focused on the radiographic assessment of permanent first molars (FPMs), as they are the first to erupt in the oral cavity at around the age of six years and are the most significant teeth from a functional and developmental perspective [9-11]. The primary cause of the loss of permanent first molars is caries [11]. Therefore, applying preventive methods requires valid data regarding the missing teeth and their cause.

Our study was focused on adults (>18 years old). To the best of our knowledge, no studies primarily focused on adult populations except for a recent clinical study [5]. However, their study focused on mandibular first permanent molars only. In general, few newly updated data are reported in the literature regarding the status of permanent first molars in Saudi Arabia, especially among adults. For example, the population in a study by Atieh MA et al. [12] was 14-19 years, in Alshamrani H. An et al. [13], it was 13-15 years, in Rezaie M et al. [14], it was 7-60 years, in Safadi R et al. [15], it was 13-20 years, in Almugla YM [7], it was 7-29 years, and in Almarghlani A [4], it was 15-18 years. Previous studies included only the young population [9,10,13], and only some included adults [7,11,12].

The results of this study indicate that the prevalence of missing FPMs in the sample population was high. The overall prevalence of missing FPMs was 43.2%, which agrees with the findings of other studies by Halicioglu K et al. [16], Rezaie M et al. [14], Safadi R et al. [15], and Almugla YM et al. [7], which were 32.3%, 40%, 31.3%, and 39.2%, respectively, and relatively lower than the study by Atieh [12], which was (57.1%). This could be due to the difference in the study methodology, as they counted the rate of the missing FPMs not individually but out of all missing teeth. However, our result was higher than the studies done by Årtun J and Thalib L. [9], Alesia K et al. [17], and Alshamrani HA et al. [13], which were (5.1%), (10.9%), and (5.2%), respectively. These differences could be attributed to the difference in the studied population, which was more towards the younger population.

The prevalence of missing teeth numbers 16, 26, 36, and 46 were 17.9%, 18.1%, 25.5%, and 23.2%, respectively, which were higher than the studies done by Saheeb et al. [18] (9% for 36 and 10.4% for 46), and Almugla YM et al. [7] who found that the prevalence of the missing teeth numbers 16, 26, 36, and 46 were 9%, 9%, 21.9%, and 19.1%, respectively. As mentioned earlier, these differences could be attributed to the difference in the study population, i.e., only adults (>18 years) were included in our study. In contrast, younger ages (7-29 years) in the study by Almugla YM et al. [7] and (>14 years) in the study by Saheeb et al. [18] were studied. The results may be explained by the fact that many people treated in a university hospital come from low socio-economic status. Similar results were found in a study by Corraini et al. [19], where teeth numbers 16, 26, 36, and 46 were missing in 18%, 19.3%, 27.7%, and 19.1% of the cases, respectively. As discussed earlier, the reason behind that could be the study population's wider age group (7 to >60 yrs).

In consistency with the literature, the distribution of missing FPMs in relation to the dental arch in the current study shows that the lower first permanent molars (LFPMs) were found missing more frequently than the upper first permanent molars (UFPMs) counterparts 16 and 26, in about 33.9% and 26.2%, respectively which was in agreement with other conducted studies by Saheeb et al. [18] (33.4% UFPMs and 45.6% LFPMs), Halicioglu K et al. [16] (15.08% UFPMs and 26.8% LFPMs), Rezaie M et al. [14] (22.6% UFPMs and 33.5% LFPMs), Safadi R et al. [15] (27.4% UFPMs and 72.6% LFPMs), and Almugla YM et al. [7] (17.4% UFPMs and 35.2% LFPMs). Moreover, according to Corraini et al. [19], Brazil's most common missing molars were LFPMs. Many factors can be associated with an increased rate of caries involving the LFPMs compared to the UFPMs, including morphology, earlier eruption times, and greater plaque accumulation in the mandibular posterior region [14].

Moreover, in the present study, the left LFPMs showed the highest number of missing FPMs (25.6%), which is comparable to the Almugla YM et al. [7] and Rezaie M et al. [14] studies, which found that the frequency of missing LFPMs in the left quadrant were 21.9% and 27.7%, respectively.

Furthermore, in terms of the prevalence of missing FPMs on the right and left sides of the dental arches, the finding of this study shows no statistically significant difference between the number of missing FPMs on the different sides of the arches (p=0.590), which is consistent with the findings of a previous study on the Saudi population [7].

Among all the age groups of our study, the prevalence of subjects with one, two, three, and four missing FPMs was 19%, 12.2%, 6.3%, and 5.5%, respectively. Similarly, a study by Rezaie M et al. [14] found that the prevalence of one, two, three, and four missing FPMs were 17.4%, 10.3%, 7.1%, and 4.9%, respectively, which is comparable to our study. Most likely due to the inclusion of older adults in the studied age group (7-75 years). The results were only seen in a study conducted by Almugla YM et al. [7], which included a narrower age range of the studied population (7-29 years). They reported that the prevalence of one, two, three, and four missing FPMs was 23.1%, 13.3%, 2.8%, and 0%, respectively, showing a higher percentage in the prevalence of one missing FPM, similar results to the prevalence of two missing FPMs, and less than the prevalence of three and four missing FPMs.These differences could be attributed to the difference in the younger age of their studied population.

Among the different types of restorations (Table 6), tooth-colored restoration was found to be the most common type of restoration among the restored FPMs, with a highly significant difference. However, major drawbacks of the tooth-colored restorations were mentioned in a systematic review, including polymerization shrinkage and polymerization stress. These have the potential to initiate failure at the composite-tooth interface, which may contribute to a rapid progression of recurrent caries. Hence, it was suggested that such restorations should be frequently followed up for early detection of failure [20]. Because the study was done retrospectively on Panoramic images. Radiographically it was not possible to distinguish the exact type of tooth-colored restoration.

The prevalence of root canal-treated teeth among the FPMs showed no statistically significant difference between the teeth (p=0.150). However, a previous study concerning the rate of endodontically treatment failure among all the types of teeth found that the most frequently treated teeth were the right lower FPM (11.3%), left lower FPM (10%), right upper FPM (7.0%), and left upper FPM (6.5%) [21].

The survivability of root canal-treated teeth restored with a crown was significantly higher than other restorations, with long-term survival of 10 years at around 81%, according to a systematic review by Stavropoulou AF [22]. In addition, it was reported that root canal-treated teeth restored with crown have a higher survival rate from fracture than resin composite [23]. In our study, only 45.5% of the root canal-treated FPMs were crowned, and 11.3% of them were left unrestored. Further results for the status of FPMs could not be compared with previous studies as those data were not evaluated in those studies. However, overall, the reasons for the loss of FPMs were found to be caries. In addition, our study's relatively higher percentages of FPMs (16-74.4%, 26-77.3%, 36-81.9%, and 37-81.9%) were either carious or restored.

In our study, the studied population was divided into three age groups. We found that the maximum number of missing FPMs were found in the 31-50 years age group and the least among the < 30 years age group. Surprisingly, there were fewer missing FPMs among the (>50 years) age group, which indicates recent changes in food habits (increased carbohydrate intake and junk food consumption) and poor oral hygiene. Among the several etiological factors contributing to teeth loss, there is a general agreement in the literature that dental caries and periodontal disease are the main reasons for teeth loss. As previous studies reported, subjects younger than 40 years of age are more prone to teeth loss due to caries, whereas periodontal disease is more common in 40 years and above age group [24]. Moreover, there are differences in the prevalence of caries across countries and with respect to geographic location, occupation, income, social class, ethnic group, education, and lifestyle.

The influence of gender on tooth loss has been reported in the literature, with a female predilection for higher rates of missing teeth [25]. However, Halicioglu et al. [16], in a study on the Turkish population aged 13-20 years, and Almugla YM et al. [7], in a study on the Saudi population aged 7-29 years, found that the gender difference in terms of missing FPMs was not significant. This study shows that 49.3% of females and 50.7% of males had at least one missing FPM, and the association between gender and missing FPM was not significant (p=0.159).

This study had some limitations, which included the nature of observational design and subjective evaluations. Nevertheless, the sample size is within relatively acceptable limits concerning the generalizability of the findings. The studied population data were retrieved from a single center. Furthermore, missing FPMs replaced with removable prostheses were considered not replaced, periodontal status was not assessed, and Temporary restorations could not be differentiated from permanent restorations radiographically. For that reason, they were grouped in one category as tooth-colored, so the status was not necessarily determining the definitive implemented treatment.

Future studies with larger sample sizes as well as the use of more objective examination methods may improve the outcome of the results and give a more precise evaluation. Further studies may be needed regarding the impact of such information on patient management and overall prognosis. We suggest conducting further studies in multiple centers throughout the country. Because of the importance of this tooth, preventive methods are highly required to maintain and minimize the mortality of FPMs. The significance of determining the status of the FPMs is to aid in the future planning of preventive and educational programs and early dental interventions to avoid complex treatments. Therefore, improving the quality of life of patients.

## Conclusions

In conclusion, a high prevalence rate of missing FPMs was found among adults in this study population. This calls for the need for dental education programs and the implementation of prevention measures to preserve such significant teeth, reduce the prevalence of loss, and minimize adverse effects and the need for complex dental procedures. Further, prospective studies on a larger population are needed to comprehensively evaluate the status of FPMs and better understand the associated factors.

## References

[1] Kurokawa M, Kanzaki H, Tokiwa H (2016). The main occluding area in normal occlusion and mandibular prognathism. Angle Orthod.

[2] Schroeder M, Schroeder D, Santos D, Leser M (2011). Molar extractions in orthodontics. Dental Press J Orthod.

[3] Demirci M, Tuncer S, Yuceokur AA (2010). Prevalence of caries on individual tooth surfaces and its distribution by age and gender in university clinic patients. Eur J Dent.

[4] Almarghlani A (2022). Prevalence, predictors, and reasons for permanent tooth extraction among high school students in Saudi Arabia: a national cross-sectional study. Cureus.

[5] Almahdi HM, Alabdrabulridha Z, AlAbbas J, Saad AA, Alarka I, Alghatm S, Alqasem H (2022). Permanent first mandibular molar: loss prevalence and pattern among Saudis in Al-Ahsa. Eur J Dent.

[6] Alhabdan YA, Albeshr AG, Yenugadhati N, Jradi H (2018). Prevalence of dental caries and associated factors among primary school children: a population-based cross-sectional study in Riyadh, Saudi Arabia. Environ Health Prev Med.

[7] Almugla YM (2021). Prevalence of missing first permanent molars in a selected population in a university dental clinic setting: a retrospective radiographic study. Int J Clin Pediatr Dent.

[8] Saunders RH Jr, Meyerowitz C (2005). Dental caries in older adults. Dent Clin North Am.

[9] Artun J, Thalib L (2011). Mesial migration and loss of first molars among young adolescents in Kuwait. Community Dent Health.

[10] Ebrahimi M, Ajami BA, Sarraf Shirazi AR, Afzal Aghaee M, Rashidi S (2010). Dental treatment needs of permanent first molars in Mashhad schoolchildren. J Dent Res Dent Clin Dent Prospects.

[11] Saber AM, Altoukhi DH, Horaib MF, El-Housseiny AA, Alamoudi NM, Sabbagh HJ (2018). Consequences of early extraction of compromised first permanent molar: a systematic review. BMC Oral Health.

[12] Atieh MA (2008). Tooth loss among Saudi adolescents: social and behavioural risk factors. Int Dent J.

[13] Al-Shammari KF, Al-Ansari JM, Al-Melh MA, Al-Khabbaz AK (2006). Reasons for tooth extraction in Kuwait. Med Princ Pract.

[14] Rezaie M, Ghapanchi J, Haghnegahdar A, Khojastehpour L, Khorshidi H, Heidari H (2018). A radiographic evaluation of missing of permanent first molars in a group of Iranian children and adults: a retrospective study. Int J Dent.

[15] Safadi R, Al-Safadi R, Al-Safadi R (2018). Prevalence of first permanent molar loss in a population of Saudi adolescents and young adults. Int J Recent Trends Sci Technol.

[16] Halicioglu K, Toptas O, Akkas I, Celikoglu M (2014). Permanent first molar extraction in adolescents and young adults and its effect on the development of third molar. Clin Oral Investig.

[17] Alesia K, Khalil HS (2013). Reasons for and patterns relating to the extraction of permanent teeth in a subset of the Saudi population. Clin Cosmet Investig Dent.

[18] Saheeb BD, Sede MA (2013). Reasons and pattern of tooth mortality in a Nigerian Urban teaching hospital. Ann Afr Med.

[19] Corraini P, Baelum V, Pannuti CM, Pustiglioni AN, Romito GA, Pustiglioni FE (2009). Tooth loss prevalence and risk indicators in an isolated population of Brazil. Acta Odontol Scand.

[20] Fernandes NA, Vally Z, Sykes LM (2015). The longevity of restorations -a literature review. J Dent Assoc S Afr.

[21] Yousuf W, Khan M, Mehdi H (2015). Endodontic procedural errors: frequency, type of error, and the most frequently treated tooth. Int J Dent.

[22] Stavropoulou AF, Koidis PT (2007). A systematic review of single crowns on endodontically treated teeth. J Dent.

[23] Jirathanyanatt T, Suksaphar W, Banomyong D, Ngoenwiwatkul Y (2019). Endodontically treated posterior teeth restored with or without crown restorations: a 5-year retrospective study of survival rates from fracture. J Investig Clin Dent.

[24] Ali D (2021). Reasons for extraction of permanent teeth in a university dental clinic setting. Clin Cosmet Investig Dent.

[25] George B, John J, Saravanan S, Arumugham IM (2011). Prevalence of permanent tooth loss among children and adults in a suburban area of Chennai. Indian J Dent Res.

